# PhylomeDB v4: zooming into the plurality of evolutionary histories of a genome

**DOI:** 10.1093/nar/gkt1177

**Published:** 2013-11-25

**Authors:** Jaime Huerta-Cepas, Salvador Capella-Gutiérrez, Leszek P. Pryszcz, Marina Marcet-Houben, Toni Gabaldón

**Affiliations:** ^1^Bioinformatics and Genomics Programme, Centre for Genomic Regulation (CRG), Dr. Aiguader, 88. 08003 Barcelona, Spain, ^2^Universitat Pompeu Fabra (UPF), 08003 Barcelona, Spain and ^3^Institució Catalana de Recerca i Estudis Avançats (ICREA), Pg. Lluís Companys 23, 08010 Barcelona, Spain

## Abstract

Phylogenetic trees representing the evolutionary relationships of homologous genes are the entry point for many evolutionary analyses. For instance, the use of a phylogenetic tree can aid in the inference of orthology and paralogy relationships, and in the detection of relevant evolutionary events such as gene family expansions and contractions, horizontal gene transfer, recombination or incomplete lineage sorting. Similarly, given the plurality of evolutionary histories among genes encoded in a given genome, there is a need for the combined analysis of genome-wide collections of phylogenetic trees (phylomes). Here, we introduce a new release of PhylomeDB (http://phylomedb.org), a public repository of phylomes. Currently, PhylomeDB hosts 120 public phylomes, comprising >1.5 million maximum likelihood trees and multiple sequence alignments. In the current release, phylogenetic trees are annotated with taxonomic, protein-domain arrangement, functional and evolutionary information. PhylomeDB is also a major source for phylogeny-based predictions of orthology and paralogy, covering >10 million proteins across 1059 sequenced species. Here we describe newly implemented PhylomeDB features, and discuss a benchmark of the orthology predictions provided by the database, the impact of proteome updates and the use of the phylome approach in the analysis of newly sequenced genomes and transcriptomes.

## INTRODUCTION

Phylogenomics—the study of genomes from an evolutionary perspective (1)—offers an ideal framework for extracting relevant biological knowledge from the continuously growing amount of available genome sequence data. For instance, the origin and evolution of relevant phenotypic features of a given group of organisms should ultimately be related to underlying genome changes, and these can be revealed by particular evolutionary patterns in the relevant gene families. Given the plurality of evolutionary histories among genes encoded in a given genome (2,3), many such approaches involve the reconstruction and analysis of large collections of phylogenetic trees. In addition, considering the broad range of available methods and the expertise needed to define an appropriate state-of-the-art phylogenetic pipeline in a fully automated way (4), many biologists benefit from the availability of pre-computed phylogenies for the genes and genomes of their interest. PhylomeDB was created in 2006 as a repository of complete collections of evolutionary histories of genes encoded in a given genome (i.e. the phylome) (5,6). It provides alignments and trees enriched with relevant annotations, as well as prediction of orthology and paralogy relationships, all of which can be searched, downloaded or visualized interactively. PhylomeDB is unique among other phylogenetic repositories (7–11), in that it follows an approach that is both gene-centric and genome-wide. In brief [for a detailed description see (6)], for each protein-coding gene (the seed gene) in a given genome (the seed genome), the PhylomeDB pipeline recapitulates the steps that a phylogeneticist will do to reconstruct the evolution of a given gene. This basically includes finding homologs in a given set of target species, which define the taxonomic scope of the phylome, aligning their sequences, filtering poorly aligned regions, selecting the most appropriate evolutionary model and building a phylogenetic tree. Each step is performed using state-of-the-art methodologies and programs. For example, alignments are generated using a combination of three different alignment programs run over the sequences in a forward and reverse orientation, i.e. the heads or tails approach (12). The information from these six different alignments is not only used to create a consistency-based consensus alignment (13) but also to inform a subsequent filtering of alignment columns containing residue pairs observed in just one underlying alignment, as implemented in trimAl version 1.4 (14). This procedure is applied sequentially to every gene in the seed genome, ensuring maximum coverage. In addition, this gene-centric approach circumvents the problems associated with defining gene families. Gene families are inherently hierarchical in nature, diversifying in complex ways due to events of gene duplication and loss (15). However, current approaches define families by clustering a network of pairwise relations to identify densely connected sub-networks that cannot represent the actual hierarchy present in the data (16). A gene-centric approach overcomes this step and results in a comprehensive collection of evolutionary histories, each one taken from the perspective of a single gene. An additional advantage of a gene-centric approach is the partial redundancy contained in the collection, with many evolutionary events captured in several trees, built from paralogous genes. This enables the use of consistency-based approaches in downstream evolutionary analyses, such as the detection of duplications (17), and the inference of orthology and paralogy relationships (18). Here we describe the main new features of PhylomeDB version 4 and discuss some recent analyses.

## AN EXPANDING PHYLOME REPOSITORY AND IMPROVED DATA ORGANIZATION

PhylomeDB is currently the largest repository of pre-computed phylogenies and provides evolutionary computations for >10 million proteins in ∼1000 species. The current release has significantly increased in size with 103 additional public phylomes, meaning roughly a 7-fold increase. In all, 42 of these new phylomes correspond to the commitment of PhylomeDB to significantly cover the reference proteomes from the quest for orthologs initiative (19,20), whereas others are the result of large-scale analyses that have been part of scientific studies or of collaborations with genome-annotation projects. Given the large amount of new phylomes stored in PhylomeDB, a set of collections has been created that group sets of phylomes. These can unite phylomes that use related organisms as seeds (e.g. Plants, Fungi, Vertebrates or Bacteria collections) or those associated to a given subject or data set (e.g. Model species, quest for orthologs reference proteomes). Collections serve to limit the scope of tree searches and to facilitate access to the relevant data to a variety of user communities, and can be accessed from a section one click away from the entry page (http://phylomedb.org/collections), which provides relevant descriptions. In addition, a new phylome search panel has been created that allows filtering phylomes by their species content and selecting several of them for the subsequent tree searches. Tree searches can be manually limited to a particular set of phylomes through the use of the ‘phyid’ parameter implemented in our URL query system (Table 1). Finally, PhylomeDB version 4 provides coherent sets of orthology and paralogy predictions using the most up-to-date release of consistency-based predictions from the MetaPhOrs database (18).

**Table 1.** List of query terms supported by the phylomeDB web API

URL query term	Value
Seqid	Any sequence identifier (i.e. Uniprot ID, Ensembl ID). Required
Phyid	A phylome ID (i.e. 102), a list of comma-separated phylome IDs or a collection ID (i.e. PhyC1). By default a tree from the most recent phylome will be selected.
Method	The preferred evolutionary model for the target tree. Default: best model.
Snode	a comma-separated list of target nodes, defined as follows: node_feature|search_pattern|fgcolor|bgcolor, where *node_feature* is one of the text-based node attributes available: name: leaf names as shown in the tips of the tree (i.e. TP53) phylomedb_name: phylomedb ID format (i.e. Phy00086SJ) gene_name: original ID used in the source proteome (i.e. ORF_1) swissprot_name: a swissprot ID (i.e. P04637) trembl_name: a trembl ID (i.e. K7PPA8) ensembl_name: any protein, transcript or gene ensembl ID (i.e. ENSP00000269305) genolevures_name: an Ascomycete-based Genolevures database ID taxid: a NCBI taxa ID (i.e. 9606) species: Uniprot species code (i.e HUMAN) spname: scientific name (i.e spiens) relative_age: any of the tracked NCBI taxa names (i.e. Primates) *search_pattern* must be a text string or a perl regular expression. *fgcolor* and *bgcolor* are optional parameters controlling foreground and background colors of the matching nodes (color should be one of the SVG color names or a RGB color code) Example: http://beta.phylomedb.org/?q=search_tree&seqid=TP53&snodes=species|MOUSE|red,best_name|TP73|blue|grey,spname|melano,relative_age|primates|blue|steelblue
Tree_features	A comma separated list of tree features to be shown. Currently the following features are supported: best_name, name, gene_name, swissprot_name, trembl_name, ensembl_name, genolevures_name, taxid, spname, lineage, motifs and support.
	Example: http://beta.phylomedb.org/?q=search_tree&seqid=TP53&tree_features=best_name,ensembl_name,spname,lineage

Only seqid is required to perform a query.

## MEETING NEW CHALLENGES: TRANSCRIPTOME-BASED PHYLOMES

PhylomeDB approach has been proven useful in the annotation and analysis of newly sequenced genomes (21–25). Including a phylogenomic approach in the genome annotation pipeline serves not only to produce a comprehensive catalog of orthology and paralogy relationships of the newly sequenced species and their relatives of interest but also for other many purposes, including, among many others, the reconstruction of the species phylogeny, or the detection of gene family expansions and contractions that may relate to the emergence of particular phenotypes. In recent years, massive transcriptome sequencing and assembly has been increasingly used as an alternative to the sequencing of whole genomes. This has been shown to be a cost-effective approach to address many functional and evolutionary questions about an organism. We have tested our pipeline and procedures in three transcriptome sets for early dipterans (26). Compared with high-coverage genomes, transcriptomes generally have more missing, incomplete and fragmented genes, a scenario that is similar to that of low-coverage genomes (27). As a result, large-scale phylogenetic data sets derived from transcriptomes are more noisy, and downstream analyses have to be carefully interpreted. In the mentioned dipteran study, we found that homolog identification was severely affected by the fragmented nature of the genes in the seed species, and thus transcriptome-based phylomes are best analyzed in conjunction with a phylome generated using a related species as a complementary seed (e.g. *Drosophila* in this case). Despite the mentioned caveats, transcriptome-based phylomes were useful as an efficient way of detecting orthologs for functional studies and for addressing phylogenetic relationships. Phylomes including transcriptome data in PhylomeDB will be tagged specifically to provide the users with the choice of using data containing transcriptomes.

## THE IMPACT OF PROTEOME UPDATES: THE HUMAN PHYLOME 6 YEARS LATER

Contrary to other databases, trees in PhylomeDB are not re-computed in each release. Phylomes are computationally demanding, and the limitation of our resources means that we face the dilemma of using them to either recompute existing phylomes or generate new ones. Newer annotations are generated rarely for most of the species considered, with the obvious exception of those from model species and those coming from constantly updated databases such as Ensembl (28). The question remains open as to which is the level of change that will render one phylome obsolete. Certainly, this depends on the desired use of the given phylome, which varies from user to user. To assess the impact of proteome updates on automatically computed phylomes, we compared two different versions of the human phylome across eukaryotes, the original one (29), published in 2007, and one re-computed 6 years later, including newer releases of all proteomes. To our knowledge this is the first time that the impact of a proteome update on a large-scale phylogenetic analysis is reported. This phylome update, including many model species and differing in 6 years of intensive research in the genome annotation field, should be considered an extreme case. For instance, Ensembl protein sets for human changed from 32 010 to 21 088 proteins (−32%), of which 13 729 were identical among both sets, and 4980 included some sort of sequence update in the newest release. Other model organisms such as *Caenorhabditis elegans* displayed larger levels of change (maintaining only ∼50% of nearly identical proteins). Seven other species such as *Saccharomyces cerevisiae* or *Tetraodon nigroviris* only changed slightly (<1% of the data set). Overall most of the species (29 of 39) retained relatively high levels (>80%) of equivalences among both sets. Because most of the changes, as shown above for human, correspond to the removal of predicted proteins, the total number of trees (19 565 versus 19 621) and the average number of proteins per tree (66 versus 65) remain stable, but result on a higher coverage over the query proteome (61 versus 93%), suggesting that most removed proteins were predicted gene models without homologs in other species. Resulting phylogenies differed by a normalized Robinson and Foulds distance of 21% different partitions, and 71% of the predicted orthologous pairs were conserved among both releases. More downstream analyses were less affected such as the reconstruction of a species tree using either a gene tree parsimony approach (14% different partitions) or the concatenation of one-to-one orthologs (8% different partitions). Changes in the final phylogeny correspond to variable positions of *Gillardia theta* and *Enzephalitozoon cuniculi*. Thus, significantly improved predicted gene sets can significantly affect downstream analyses to various degrees, and thus phylome updates would be recommended after major releases of the seed proteome or of several of the other proteomes included. Three of the oldest phylomes have been re-computed using largely updated proteomes: the aforementioned human phylome, those of *Schistosoma mansoni* (30) and *Acyrthosyphon pisum* (22). We will be constantly monitoring the need to recompute phylomes based on the availability of significantly changed proteomes and the general use of the existing phylome. We encourage users to suggest updates, whenever significant re-annotations are performed. Deprecated phylomes will still be available for download.

## BENCHMARKING ORTHOLOGY IN THE QUEST FOR ORTHOLOGS INITIATIVE

The quest for orthologs initiative aims at sharing knowledge among users and developers of orthology prediction algorithms and databases, as well as establishing standards in this field (19,20). One of the main achievements so far has been the development of a common benchmarking resource, gathering some of the available tests assessing different properties of predicted orthologous sets (31,32). Although existing benchmarks represent a necessarily indirect way of measuring accuracy of orthology prediction, and are difficult to interpret, they nevertheless represent a useful tool for algorithm developers, who now can observe and understand the behavior of their algorithms on different data sets, tests and parameter sets. We took this opportunity to test a few parameters of our orthology prediction algorithm. Our algorithm is based on the concept of detecting duplications using a species-overlap threshold (16,29). We investigated the accuracy of pairwise predictions in relation to the nodal distance to the seed. We found that drawing predictions of pairwise relationships only for pairs of sequences including at least one pertaining to the subset of 30 sequences closest to the seed (close2seed) improved sensitivity (15% for agreement with reference phylogeny test), without significantly altering specificity. This is because for large phylogenies with multiple paralogs, less reliable signal from collateral trees —i.e. those in which the sequence is present but not used as a seed—would overcome the signal from seed trees. For our data sets, using close2seed of 30, performed the best, and therefore this has been implemented in the default orthology prediction algorithm. Overall orthology predictions from PhylomeDB obtained a good compromise between coverage and accuracy in the different benchmarks (http://orthology.benchmarkservice.org). For instance, in a benchmark based on the agreement with a reference tree of eukaryotes (32), PhylomeDB provided 11 872 orthology predictions and trees reconstructed from these orthologs differed from the reference species topology by 6.7% partitions, on average.

## NEW DATA AND VISUALIZATION FEATURES

**Figure 1.**
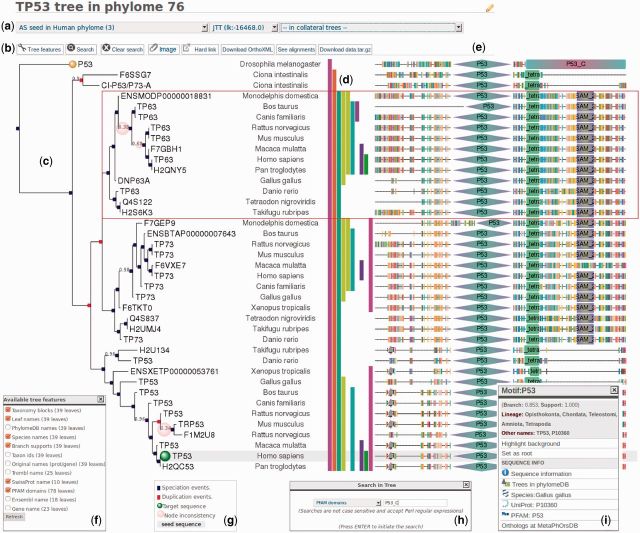
Example of the integrated tree visualization interface showing the gene family phylogeny of TP53. (**a**) The tree search panel allows switching among all available trees containing the target sequence, even if it was not used as a seed (i.e. collateral tree). (**b**) The tree editing menu allows to search nodes matching custrom criteria, select what tree features are shown in the image and download image or other data. (**c**) Lowly supported nodes are highlighted with a transparent bubble and speciation and duplication events are indicated using red and blue colors, respectively. (**d**) A taxonomy panel indicating the assignment of different partitions to major taxonomic levels. Taxonomic level associated to each color is shown on mouse over events. (**e**) Domain and sequence panel. PFAM motifs are represented by different shapes and can be clicked for extended information. Inter-domain coding regions are shown using the standard amino acid color codes. Gap regions are illustrated as a flat line. (**f**) Available tree features. One or more attributes are allowed to be selected to modify the default aspect of the tree image. (**g**) The tree legend indicating color codes of the different tree nodes. (**h**) The search panel allows to search for node matching any custom criteria of a number of node attributes. In the example shown, a node containing the P53_C domain has been highlighted through the use of this panel. (**i**) The contextual node menu, including extended information about a node and links to external data source.

PhylomeDB version 4 incorporates new enhancements in tree visualization, phylogeny annotation and tree search engine. First, the backend of the tree searching engine has been improved to provide a gene-centric view of all phylomeDB resources (Figure 1a). Thus, after a protein or gene search, all the available trees in phylomeDB are listed and organized by phylome and tree type. Users can switch among all available seed and collateral trees without missing the focus on the searched protein or gene. Users can download relevant data, including the whole database, a specific phylome or, from the tree entry page, the relevant data corresponding to that tree. In this new release, we have implemented the possibility to download orthology predictions from a tree in the recently developed OrthoXML standard format (33) (Figure 1b), in addition to a tabulated format. Second, all the information available for each tree is now shown using an integrated layout in which tree topology (Figure 1c), taxonomic data (Figure 1d), alignments and domain annotations (Figure 1e) and event-age (phylostratigraphy) information are rendered in the same figure using the newest visualization features provided by the ETE toolkit version 2.2 (34): (i) PFAM domains (35) have been mapped to each alignment in our database and are now displayed in a compact panel at the right side of the tree (Figure 1e). For each sequence, domains and their names are shown; they can be clicked to obtain a short description and the external link to PFAM (Figure 1i). Protein regions not mapped to domains are shown using the standard amino acid color codes, whereas gap regions are represented by a flat line. (ii) A taxonomy-information panel has been added to the right side of every tree that allows to highlight the main taxonomic clades present within each gene tree (Figure 1d). Information on the estimated relative age (i.e. phylostratygraphy) of each tree node (17), extended taxonomic information and functional GO-term annotations (36) is provided by the contextual menu obtained when clicking on any node. (iii) Tree images have been also simplified to improve readability. Mappings and/or cross-linking to general and organism-oriented databases has been extended to include the major *Arabidopsis thaliana* sequence database TAIR (37), *Drosophila*’s Flybase (38), Candida genome database (39) and the Ascomycete-based genome database Genolevures (40). By default a single sequence identifier is shown on the tree, prioritizing those that are more suited for human interpretation, but this can be adjusted through the tree editing menu (Figure 1f). A conversion table among PhylomeDB unique identifiers and other identifiers is provided in the download section. Speciation and duplication nodes are indicated using different colors, and branch support values are now automatically highlighted for lowly supported partition using a transparent red bubble inversely proportional to the branch bootstrap or aLRT value (Figure 1g). Internal tree searches can be performed for any of the annotated node attributes (Figure 1h), whereas links to other databases are provided through the contextual menu of the tree browser that appears when clicking any node (Figure 1i), which facilitates functional inference across members of a gene family. Finally, the web-based linking API has been improved and it now allows for direct links to trees and phylomes, as well as highlighting custom nodes within a tree topology (Figure 1f). The URL format used by the API is detailed in Table 1.

## FUNDING

Spanish ministry of Economy and Competitiveness [BIO2012-37161]; a Grant from the Qatar National Research Fund [NPRP 5-298-3-086]; a the European Research Council under the European Union's Seventh Framework Programme [FP/2007-2013/ERC and ERC-2012-StG-310325]; Juan de La Cierva postdoctoral program (to J.H.C.) and La Caixa-CRG International Fellowship Program (to L.P.P.). Funding for open access charge: Internal budget from the CRG.

*Conflict of interest statement*. None declared.

## References

[1] Eisen JA, Fraser CM (2003). Phylogenomics: intersection of evolution and genomics. Science.

[2] Castresana J (2007). Topological variation in single-gene phylogenetic trees. Genome Biol..

[3] Marcet-Houben M, Gabaldón T (2009). The tree versus the forest: the fungal tree of life and the topological diversity within the yeast phylome. PLoS One.

[4] Anisimova M, Liberles DA, Philippe H, Provan J, Pupko T, von Haeseler A (2013). State-of the art methodologies dictate new standards for phylogenetic analysis. BMC Evol. Biol..

[5] Huerta-Cepas J, Bueno A, Dopazo J, Gabaldón T (2008). PhylomeDB: a database for genome-wide collections of gene phylogenies. Nucleic Acids Res..

[6] Huerta-Cepas J, Capella-Gutierrez S, Pryszcz LP, Denisov I, Kormes D, Marcet-Houben M, Gabaldon T (2011). PhylomeDB v3.0: an expanding repository of genome-wide collections of trees, alignments and phylogeny-based orthology and paralogy predictions. Nucleic Acids Res..

[7] Vilella AJ, Severin J, Ureta-Vidal A, Heng L, Durbin R, Birney E (2009). EnsemblCompara GeneTrees: complete, duplication-aware phylogenetic trees in vertebrates. Genome Res..

[8] Ruan J, Li H, Chen Z, Coghlan A, Coin LJ, Guo Y, Heriche JK, Hu Y, Kristiansen K, Li R (2008). TreeFam: 2008 Update. Nucleic Acids Res..

[9] Mi H, Muruganujan A, Thomas PD (2013). PANTHER in 2013: modeling the evolution of gene function, and other gene attributes, in the context of phylogenetic trees. Nucleic Acids Res..

[10] Afrasiabi C, Samad B, Dineen D, Meacham C, Sjolander K (2013). The PhyloFacts FAT-CAT web server: ortholog identification and function prediction using fast approximate tree classification. Nucleic Acids Res..

[11] Penel S, Arigon AM, Dufayard JF, Sertier AS, Daubin V, Duret L, Gouy M, Perriere G (2009). Databases of homologous gene families for comparative genomics. BMC Bioinformatics.

[12] Landan G, Graur D (2007). Heads or tails: a simple reliability check for multiple sequence alignments. Mol. Biol. Evol..

[13] Wallace IM, O'Sullivan O, Higgins DG, Notredame C (2006). M-Coffee: combining multiple sequence alignment methods with T-Coffee. Nucleic Acids Res..

[14] Capella-Gutierrez S, Silla-Martinez JM, Gabaldón T (2009). trimAl: a tool for automated alignment trimming in large-scale phylogenetic analyses. Bioinformatics.

[15] Gabaldon T, Koonin EV (2013). Functional and evolutionary implications of gene orthology. Nat. Rev. Genet..

[16] Gabaldón T (2008). Large-scale assignment of orthology: back to phylogenetics?. Genome Biol..

[17] Huerta-Cepas J, Gabaldón T (2010). Assigning duplication events to relative temporal scales in genome-wide studies. Bioinformatics.

[18] Pryszcz LP, Huerta-Cepas J, Gabaldon T (2010). MetaPhOrs: orthology and paralogy predictions from multiple phylogenetic evidence using a consistency-based confidence score. Nucleic Acids Res..

[19] Gabaldon T, Dessimoz C, Huxley-Jones J, Vilella AJ, Sonnhammer EL, Lewis S (2009). Joining forces in the quest for orthologs. Genome Biol..

[20] Dessimoz C, Gabaldón T, Roos DS, Sonnhammer EL, Herrero J (2012). Toward community standards in the quest for orthologs. Bioinformatics.

[21] Marcet-Houben M, Ballester AR, de la Fuente B, Harries E, Marcos JF, Gonzalez-Candelas L, Gabaldon T (2012). Genome sequence of the necrotrophic fungus Penicillium digitatum, the main postharvest pathogen of citrus. BMC Genomics.

[22] Huerta-Cepas J, Marcet-Houben M, Pignatelli M, Moya A, Gabaldón T (2010). The pea aphid phylome: a complete catalogue of evolutionary histories and arthropod orthology and paralogy relationships for Acyrthosiphon pisum genes. Insect Mol. Biol..

[23] Collen J, Porcel B, Carre W, Ball SG, Chaparro C, Tonon T, Barbeyron T, Michel G, Noel B, Valentin K (2013). Genome structure and metabolic features in the red seaweed Chondrus crispus shed light on evolution of the Archaeplastida. Proc. Natl Acad. Sci. USA.

[24] Garcia-Mas J, Benjak A, Sanseverino W, Bourgeois M, Mir G, Gonzalez VM, Henaff E, Camara F, Cozzuto L, Lowy E (2012). The genome of melon (Cucumis melo L). Proc. Natl Acad. Sci. USA.

[25] Peña A, Teeling H, Huerta-Cepas J, Santos F, Yarza P, Brito-Echeverria J, Lucio M, Schmitt-Kopplin P, Meseguer I, Schenowitz C (2010). Fine-scale evolution: genomic, phenotypic and ecological differentiation in two coexisting Salinibacter ruber strains. ISME J..

[26] Jimenez-Guri E, Huerta-Cepas J, Cozzuto L, Wotton KR, Kang H, Himmelbauer H, Roma G, Gabaldon T, Jaeger J (2013). Comparative transcriptomics of early dipteran development. BMC Genomics.

[27] Milinkovitch MC, Helaers R, Depiereux E, Tzika AC, Gabaldon T (2010). 2X genomes— depth does matter. Genome Biol..

[28] Flicek P, Amode MR, Barrell D, Beal K, Brent S, Carvalho-Silva D, Clapham P, Coates G, Fairley S, Fitzgerald S (2012). Ensembl 2012. Nucleic Acids Res..

[29] Huerta-Cepas J, Dopazo H, Dopazo J, Gabaldón T (2007). The human phylome. Genome Biol..

[30] Silva LL, Marcet-Houben M, Nahum LA, Zerlotini A, Gabaldon T, Oliveira G (2012). The Schistosoma mansoni phylome: using evolutionary genomics to gain insight into a parasite's biology. BMC Genomics.

[31] Boeckmann B, Robinson-Rechavi M, Xenarios I, Dessimoz C (2011). Conceptual framework and pilot study to benchmark phylogenomic databases based on reference gene trees. Brief. Bioinform..

[32] Altenhoff AM, Dessimoz C (2009). Phylogenetic and functional assessment of orthologs inference projects and methods. PLoS Comput. Biol..

[33] Schmitt T, Messina DN, Schreiber F, Sonnhammer EL (2011). Letter to the editor: SeqXML and OrthoXML: standards for sequence and orthology information. Brief. Bioinform..

[34] Huerta-Cepas J, Dopazo J, Gabaldón T (2010). ETE: a python environment for tree exploration. BMC Bioinformatics.

[35] Punta M, Coggill PC, Eberhardt RY, Mistry J, Tate J, Boursnell C, Pang N, Forslund K, Ceric G, Clements J (2012). The Pfam protein families database. Nucleic Acids Res..

[36] Barrell D, Dimmer E, Huntley RP, Binns D, O'Donovan C, Apweiler R (2009). The GOA database in 2009—an integrated gene ontology annotation resource. Nucleic Acids Res..

[37] Lamesch P, Berardini TZ, Li D, Swarbreck D, Wilks C, Sasidharan R, Muller R, Dreher K, Alexander DL, Garcia-Hernandez M (2012). The arabidopsis information resource (TAIR): improved gene annotation and new tools. Nucleic Acids Res..

[38] Marygold SJ, Leyland PC, Seal RL, Goodman JL, Thurmond J, Strelets VB, Wilson RJ (2013). FlyBase: improvements to the bibliography. Nucleic Acids Res..

[39] Inglis DO, Arnaud MB, Binkley J, Shah P, Skrzypek MS, Wymore F, Binkley G, Miyasato SR, Simison M, Sherlock G (2012). The Candida genome database incorporates multiple Candida species: multispecies search and analysis tools with curated gene and protein information for Candida albicans and Candida glabrata. Nucleic Acids Res..

[40] Martin T, Sherman DJ, Durrens P (2011). The genolevures database. C R Biol..

